# Short Term Morphine Exposure *In Vitro* Alters Proliferation and Differentiation of Neural Progenitor Cells and Promotes Apoptosis via Mu Receptors

**DOI:** 10.1371/journal.pone.0103043

**Published:** 2014-07-29

**Authors:** Dafna Willner, Ayelet Cohen-Yeshurun, Alexander Avidan, Vladislav Ozersky, Esther Shohami, Ronen R. Leker

**Affiliations:** 1 Department of Anesthesia and Critical Care Medicine, Hadassah-Hebrew University Medical Center, Jerusalem, Israel; 2 Department of Pharmacology, Institute of Drug Research, School of Pharmacy, Hebrew University, Jerusalem, Israel; 3 Department of Anesthesia and Critical Care Medicine, Hadassah -Hebrew University Medical Center, Hebrew University School of Medicine, Jerusalem, Israel; 4 Department of Neurology, Peritz and Chantal Scheinberg Cerebrovascular Research Laboratory, Hadassah-Hebrew University Medical Center, Hebrew University School of Medicine, Jerusalem, Israel; Universitat Pompeu Fabra, Spain

## Abstract

**Background:**

Chronic morphine treatment inhibits neural progenitor cell (NPC) progression and negatively effects hippocampal neurogenesis. However, the effect of acute opioid treatment on cell development and its influence on NPC differentiation and proliferation *in vitro* is unknown. We aim to investigate the effect of a single, short term exposure of morphine on the proliferation, differentiation and apoptosis of NPCs and the mechanism involved.

**Methods:**

Cell cultures from 14-day mouse embryos were exposed to different concentrations of morphine and its antagonist naloxone for 24 hours and proliferation, differentiation and apoptosis were studied. Proliferating cells were labeled with bromodeoxyuridine (BrdU) and cell fate was studied with immunocytochemistry.

**Results:**

Cells treated with morphine demonstrated decreased BrdU expression with increased morphine concentrations. Analysis of double-labeled cells showed a decrease in cells co-stained for BrdU with nestin and an increase in cells co-stained with BrdU and neuron-specific class III β-tubuline (TUJ1) in a dose dependent manner. Furthermore, a significant increase in caspase-3 activity was observed in the nestin- positive cells. Addition of naloxone to morphine-treated NPCs reversed the anti-proliferative and pro-apoptotic effects of morphine.

**Conclusions:**

Short term morphine exposure induced inhibition of NPC proliferation and increased active caspase-3 expression in a dose dependent manner. Morphine induces neuronal and glial differentiation and decreases the expression of nestin- positive cells. These effects were reversed with the addition of the opioid antagonist naloxone. Our results demonstrate the effects of short term morphine administration on the proliferation and differentiation of NPCs and imply a mu-receptor mechanism in the regulation of NPC survival.

## Introduction

Recent evidence suggests that exposure of children to anesthesia during the prenatal and neonatal periods and early childhood may have significant influences on behavior and cognition [1], [2]. N-methyl-D-asparate (NMDA) receptor antagonists and gamma-aminobutyric acid (GABA) receptor agonists have been shown to induce neuronal injury and apoptosis in the developing brains of rodents [3]–[5]. Barbituates, benzodiazipines, inhaled anesthetics, nitrous oxide, ketamine, etomidate, and propofol mediate their drug effect via one or both of these receptors and may therefore be neurotoxic to the developing brain. The neurotoxic effects seem to result from apoptotic neurodegeneration [6], [7]. Although significant research has focused on these two mediator pathways, surprisingly, the effects of opioids, the most common analgesic used in anesthetic practice, have not been thoroughly investigated in the developing brain. Anesthetic research has only recently delved into the possible neurotoxic effects of opioids and research has predominantly focused on chronic exposure in animal models and not on the developmental impact of short term exposure to such drugs during early brain formation [8], [9].

Opioids were the first abused drug shown to negatively impact proliferation and neurogenesis in the adult mammalian hippocampus [10], [11]. Studies have demonstrated that opioid receptor antagonists, opioid peptides, and opiate drugs can all influence numerous stages in brain development, including neuronal and glial proliferation, differentiation [12], [13], and cell death [14]. Chronic morphine administration has been shown to impede various processes of brain development in rodents, including DNA synthesis [15] and alters the number of cortical neurons [16]. Neuronal cell cultures treated with morphine for 7 days showed decreased viability and increased caspase-3 activity [17]. More so, the effects of long term opioid abuse in expectant mothers have been well publicized in the literature [18].

The mu-opioid receptor (MOR) was discovered on proliferating radial glia, a neuronal progenitor cells of the CNS [19], [20], in mice [21], [22] and human neural progenitors of the subventricular zone (SVZ) [23]. Stimulation of the receptor by opioids might thereby considerably affect the behavior of cortical progenitor cells. Endogenous opioids and synthetic agonists have been shown to inhibit proliferation and differentiation of several neural cell types in both *in vivo* and *in vitro* models [8], [15], [24]. Naloxone, a non-selective, short-acting opioid receptor antagonist, competitively inhibits the pharmacologic effects of opioids. It is used extensively in clinical practice for the treatment of opioid overdose and is presently considered a safe drug over a wide dose range [25] (up to 10 mg). Morphine reduces astrocyte number and proliferation in a dose dependent manner and also affects the differentiation of neural progenitors into astrocytes [26]. The concurrent administration of naloxone resulted in a reversal of morphine's negative effect. Furthermore, morphine has been shown to have a large therapeutic window for toxic and neurodevelopmental effects [27], [28].

If these changes are observed in the mature brain, what then are the implications on the developing, immature brain where neuronal proliferation and differentiation is more extensive? More so, during the critical period of human brain development and maturation, exceedingly multi-faceted and fragile processes transpire that are vulnerable to any form of disruption, even a single drug exposure. Pregnant patients are exposed to short term opioid treatment during non-obstetric surgical procedures, with a frequency of 0.75%–2%. The majority of these procedures are performed during the first and second trimester of pregnancy (42% and 35%, respectively) [29]. We, therefore, decided to study the impact of short term morphine exposure on neural progenitor cell (NPC) proliferation and differentiation in the developing brain and investigate the receptor- mediated pathway involved.

## Materials and Methods

### Cell cultures

This study was carried out in strict accordance with the recommendations in the Guide for the Care and Use of Laboratory Animals of the National Institutes of Health. The protocol was approved by the Committee on the Ethics of Animal Experiments of the Hebrew University (Permit Number: MD-12-13190-3). All surgery was performed under isoflurane anesthesia, and all efforts were made to minimize suffering. Cerebral cortical cultures were prepared from 14 day ICR mouse embryos as previously described [30]. Briefly, brains were removed and the cortices were separated from the hemispheres and added to 0.025% trypsin solution for 10 min at 37°C humidified 5% CO_2_ incubator. After removal of trypsin, tissues were added to MEM supplemented with 10% horse serum, 2 mM L-glutamine, 0.35% glucose and 0.5% penicillin/streptomycin and titrated with a pasteur pipette until a clear solution was obtained. Following centrifugation for 10 min at 1100 rpm (108 g), cells were plated onto poly-l-ornithine pre-coated 12 mm coverslips (2.0×10^5^). Cells were cultured in Neurobasal medium (Rhenium, Israel) containing 2% B27 supplement, 2 mM glutamate, and 1% penicillin/streptomycin and incubated at 37°C in a humidified 5% CO_2_ incubator. Neurobasal medium supports maintenance of populations of NPC and neuronal cells [31].

### Opioid treatments

On Day 3 (3 Days in vitro, DIV), culture wells were divided into untreated and treatment groups. For each treatment group, 4 pregnant mice were used. Morphine sulfate (RAFA laboratories, Israel) and naloxone hydrochloride (Mylan, U.S.A) were used in the treatment groups. Growth media was removed from each well and replaced with one of the following treatments for 0–24 hours: 0 morphine (control- untreated group), 0.13 µM (0.1 µg/ml) morphine, 1.3 µM (1 µg/ml) morphine, 13 µM (10 µg/ml) morphine, 13 µM morphine together with 50 µM (20 µg/ml) naloxone, and 50 µM naloxone alone dissolved in Neurobasal medium (2 ml). These doses were chosen based on preliminary experiments performed in our laboratory and on existing data in the literature [32], [33], [17]. Additionally, bromodeoxyuridine (BrdU, 25 µg/well; Sigma, Israel) was added to all treatment groups for 24 hours at this time (3DIV). Cultures were incubated at 37°C in humidified 95% air/5% CO_2_ and high humidity. After 24 hours (4 DIV) cells were returned to the normal feeding medium. Cell fixation was performed on 7 DIV with 4% paraformaldehyde.

### Apoptosis

For detection of apoptotic cells, cells were fixed in 4% paraformaldehyde on 4 DIV. Caspase-3, a proteolytic enzyme critical in the apoptotic process [34], was used for the identification of apoptotic cells. Apototic cells were detected using immunocytochemistry with the following primary and secondary antibodies, respectively: polyclonol rabbit anti-active caspase-3 (1∶200) (R&D Systems, Israel) and Alexa Fluor 555 donkey anti-rabbit IgG (1∶200) (Invitrogen, Israel).

### Immunoflourescent analysis of differentiated cells

Cell cultures were fixed on 7 DIV and stained for the proliferation marker BrdU together with one of the following fate-specific antibody markers: nestin for neuroepithelial cells/NPCs, TUJ1 (neuron-specific class III β-tubuline) for neurons, GFAP (glial fibrillary acidic protein) for astrocytes, Gal-C (galactocerebroside) for oligodendrocytes, and NeuN (neuronal nuclear antigen) for mature neurons. Cultures were incubated overnight at 4°C with one or more of the following primary antibodies: rat monoclonal anti-Brdu (1∶200) (Abcam, Israel), rabbit polyclonal anti-nestin (1∶100) (Abcam, Israel), mouse monoclonal Anti-beta Tubulin III(1∶2000) (Sigma, Israel), rabbit polyclonal anti-galactocerebroside (1∶200) (Abcam, Israel), rabbit polyclonal anti-glial fibrillary acidic protein (GFAP) (1∶200) (Dako, Israel) and mouse anti-neuronal nuclear protein (NeuN) (1∶500) (Abcam, Israel). Alexa Fluor antibodies (all 1∶200, Invitrogen, Israel) were used for secondary staining. Controls for non-specific binding included omitting primary or secondary antibodies. Specificity of BrdU immuno-staining was verified by the absence of labeling from brain cultures that did not receive BrdU. Coverslips were mounted with Vectashield (Vector) and 4′6-diamidino-2-phenylindole (DAPI) was used to counter stain nuclei.

Cells were counted in six fields per coverslip (center (x2) and 3, 6, 9, 12 o'clock). Quantitative measurements of the markers were performed using a fluorescent microscope (Olympus BX51, PA, USA) using a 40X objective equipped with a digital camera (Nikon DXM1200F, Tokyo, Japan) employing stereological methods.

### Experimental paradigm

Four pregnant mice were used in the experimental protocol. Cells from each litter were divided into four treatment groups (0 morphine (control- untreated group), 0.13 µM morphine, 1.3 µM morphine and 13 µM morphine) and plated on coverslips. In each treatment group, immunostaining for each of the following markers was performed: nestin, GFAP and TUJ1 with BrdU and caspase-3, and caspase-3 with BrdU. Eight coverslips per marker for each treatment group were attained per litter. An additional 4 pregnant mice were used in the second experimental group (0 morphine (control- untreated group), 13 µM morphine, 13 µM morphine together with 50 µM naloxone, and 50 µM naloxone alone). In addition, eight coverslips per marker for each treatment group were attained per litter. Cell dissection, tissue processing and fixation from each pregnant mouse were performed on separate days. Fresh growth media and factors were prepared for each dissection.

### Statistical data analysis

To maintain unbiased standards, histological data was obtained by investigators blinded to the treatment protocol. Statistics was evaluated with the SigmaStat 2.03 software (SPSS, Inc., Chicago, Illinois). Comparisons between groups were performed with the analysis of variance (ANOVA) followed by the Tukey post-hoc test. *P* values less than 0.05 were considered statistically significant.

## Results

### Morphine decreases proliferation of NPCs and induces the apoptotic enzyme caspase-3 in a dose dependent manner

**Figure 1:**
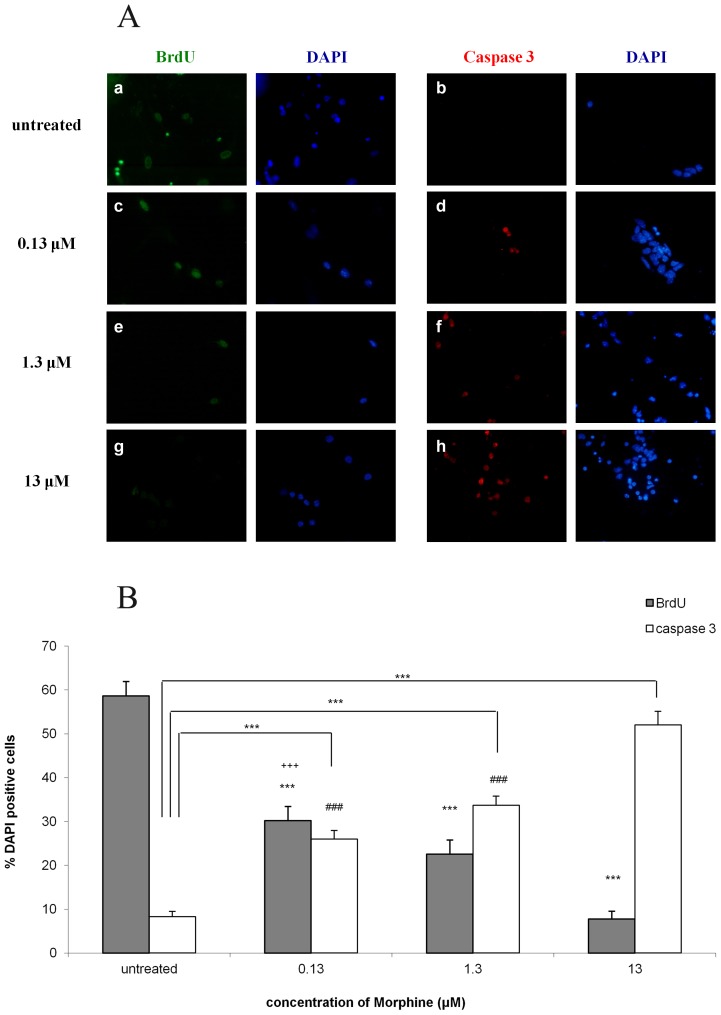
Morphine decreases proliferation of NPCs and induces the apoptotic enzyme active caspase-3 in a dose dependent manner. NPCs were exposed to increasing doses of morphine. This increase caused a decrease in the number of cells expressing BrdU and an increase in the number of apoptotic cells expressing active caspase-3 (**1A**). Bar graph showing, in a dose-dependent manner, the decrease in BrdU and increase in active caspase-3 expression in NPCs exposed to a single-dose of morphine (***p<0.001 different morphine doses vs. untreated in BrdU and caspase-3; ^###^p<0.001 0.13 µM and 1.3 µM vs. 13 µM in caspase-3; ^+++^p<0.001 0.13 µM vs. 13 µM in BrdU) (**1B**).

Fetal mouse cortical cells treated with morphine demonstrated a dose dependent decrease in BrdU incorporation compared with the untreated group (58.7±3.3% of the cells in the untreated cultures incorporated BrdU). Of the cells treated with 0.13 µM morphine, 30.2±3.2% were BrdU- positive and 22.6±3.3% and 7.8±1.8% BrdU- positive cells were observed with 1.3 µM and 13 µM morphine, respectively (p<0.001) (Figure 1A, 1B). In parallel, a significant increase in the levels of the pro-apoptotic enzyme active caspase-3 (Figure 1A, 1B) was detected (8.3±1.2% vs. 26.0±2.0%, 33.7±2.1% and 52.0±3.1% for untreated vs. 0.13 µM, 1.3 µM and 13 µM morphine-treated groups, respectively; p<0.001 for all).

### Morphine increases the levels of active caspase-3 in proliferating cells

**Figure 2:**
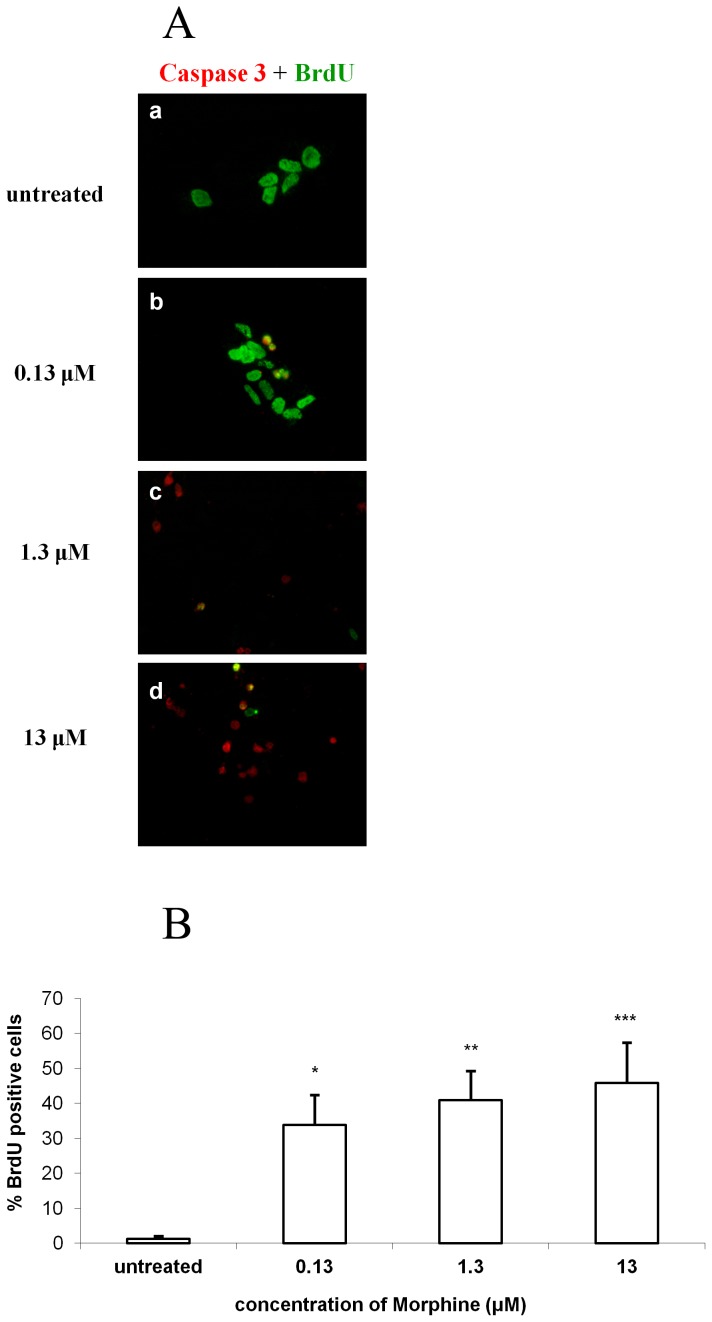
Morphine increases the levels of active caspase-3 in proliferating cells. Addition of morphine to the cultures of NPCs results in an increase in apoptosis of BrdU-positive cells with increased morphine concentrations. (*p<0.05, **p<0.01, ***p<0.001 vs. untreated) (**2A, 2B**).

We searched for a correlation between the reduction in BrdU incorporation and the increase in active caspase-3 expression. Co-staining for BrdU and caspase-3 showed that a substantial number of the proliferating cells (BrdU- positive cells) also expressed the pro-apoptotic enzyme active caspase-3. The number of double-positive cells was significantly increased in the morphine- treated groups (33.8±8.6%, 40.9±8.3% and 45.8±10.7% for 0.13 µM, 1.3 µM and 13 µM morphine, respectively) compared with the untreated group (p<0.05, p<0.01, and p<0.001, respectively, vs. untreated; Figure 2).

### Morphine increases neuronal differentiation and inhibits self-renewal of NPCs

**Figure 3:**
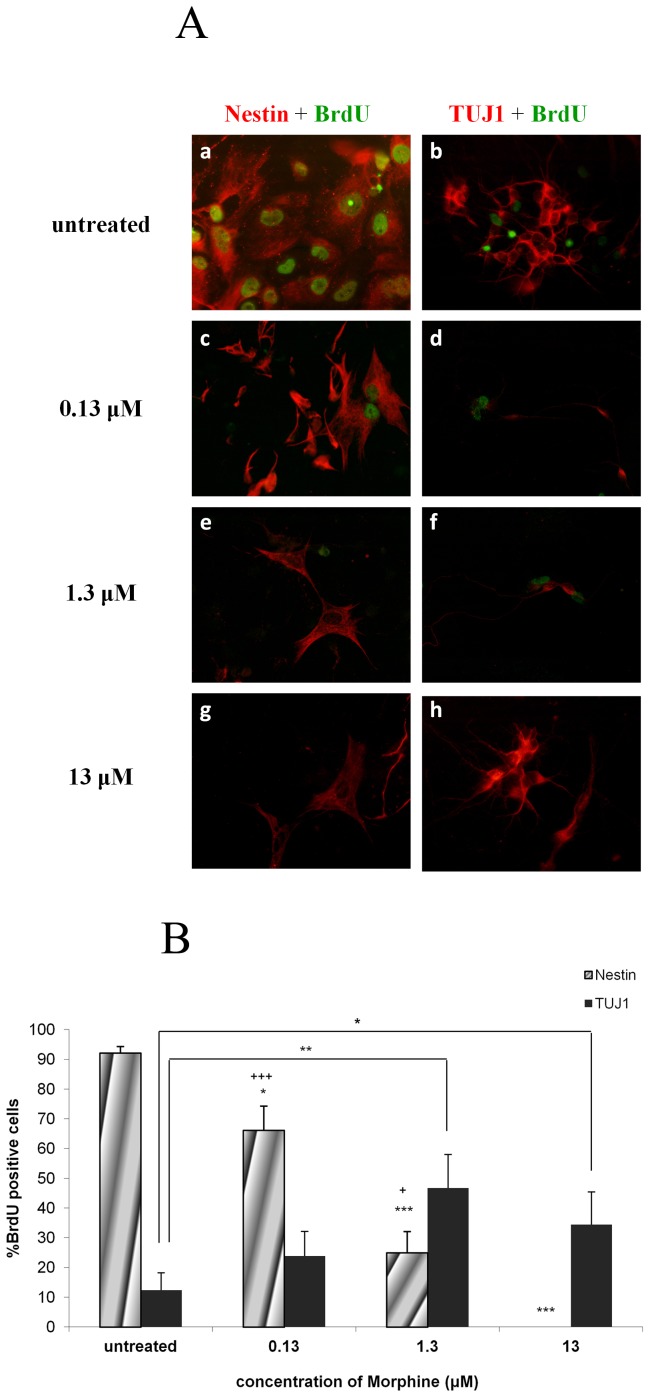
Morphine increases neuronal differentiation and inhibits self-renewal of NPCs. NPCs exposed to increasing levels of morphine demonstrated a decrease in BrdU- positive- nestin-positive cell expression while TUJ1- positive- BrdU- positive co-staining was enhanced with increased morphine concentrations (*p<0.05, **p<0.01, ***p<0.001 vs. untreated; ^+^p<0.05, ^+++^p<0.001 vs. 13 µM) (**3A, 3B**).

A dose dependent decrease in the levels of the neuroepithelial marker, nestin, was detected in cell cultures exposed to different concentrations of morphine. Untreated cultures demonstrated almost a complete profile of neuroepithelial cells, with 92.1±2.2% of cells incorporating BrdU being nestin- positive. However, in the 0.13 µM and 1.3 µM morphine-treated group, 66.1±8.1% and 24.9±7.2% of the cells, respectively, were BrdU- nestin- positive (p<0.05 and p<0.001 vs. untreated, respectively). The sharpest reduction in BrdU- nestin- positive cells was demonstrated in the cells treated with 13 µM morphine where no neuroepithelial cells from the BrdU- positive population were detected (p<0.001) (Figure 3A, 3B). No positive immunostaining was observed for oligodentrocytes (Gal C) and mature neurons (NeuN) in the untreated group as well as in all the treatment groups.

Morphine not only appears to enhance apoptosis of proliferating cells as seen in the earlier results of this work, but also may induce differentiation of cells incorporating BrdU. A significant increase in the percentage of BrdU- positive cells expressing the neuronal marker TUJ1 was observed in the cultures exposed to different concentrations of morphine in a dose dependent manner (Figure 3A, 3B).

### Morphine causes increased caspase-3 activity in NPCs and astrocytes but not in TUJ1-expressing neurons

**Figure 4:**
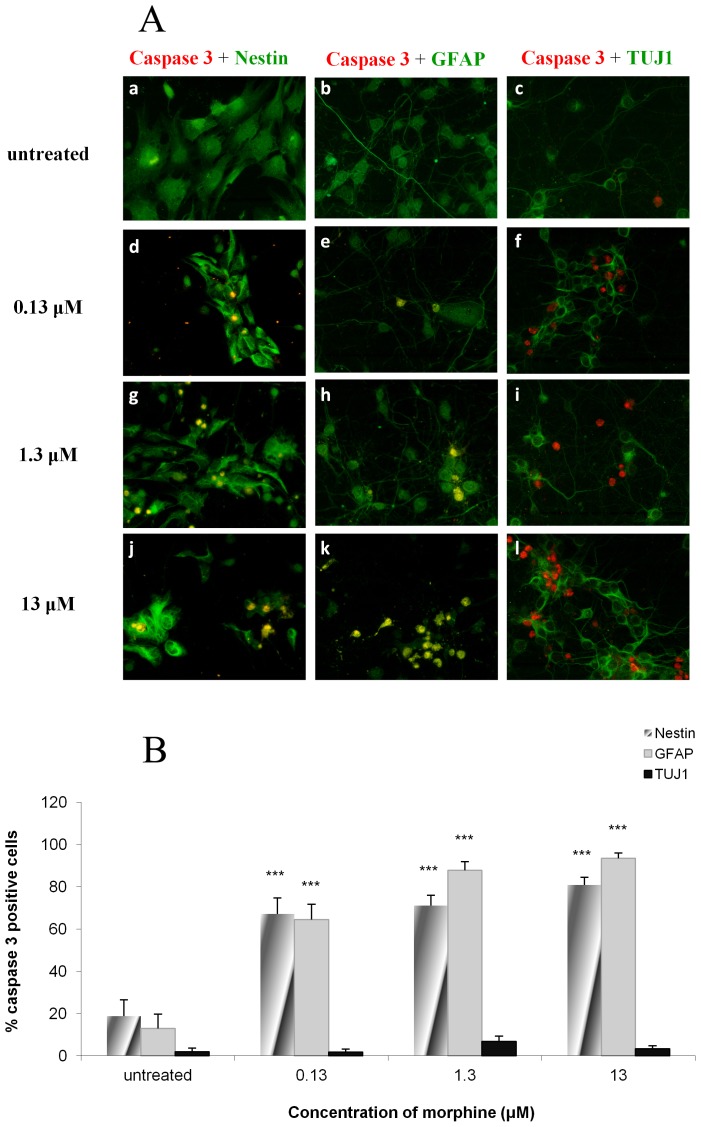
Morphine causes increased caspase-3 activity in NPCs and astrocytes but not in TUJ1-expressing neurons. NPCs expressing caspase-3 were co-stained with nestin, GFAP or TUJ1. Most of the cells expressing active caspase-3 were nestin- positive cells and GFAP- positive cells while relatively low levels of the cells were TUJ1- positive cells. The percentage of nestin- positive and GFAP- positive cells expressing caspase-3 increased in a dose dependent manner (***p<0.001 vs. untreated) (**4A, 4B**).

To identify which cells are most sensitive to the apoptotic effects of morphine, we co-stained active caspase-3 with each of the markers characterizing neuroepithelial cells, astrocytes and neurons. A substantial increase in the levels of active caspase-3- positive cells expressing nestin were detected in cultures treated with 0.13, 1.3 and 13 µM of morphine (3.6, 3.8 and 4.3-fold increase, respectively) compared with control values (Figure 4A, 4B). Similarly, a 5.0, 6.8 and up to 7.2-fold increase, respectively, was detected in the levels of active caspase-3- GFAP- positive cells in the different morphine-treatment groups (Figure 4A, 4B). No significant changes in the expression of active caspase-3 was observed in the TUJ1- positive cells treated with 0.13, 1.3 and 13 µM of morphine (Figure 4A, 4B) and no changes were observed in the overall number of TUJ1- positive- BrdU- negative cells (data not shown).

### Naloxone reverses the anti-proliferative and pro-apoptotic effects of morphine on NPCs

**Figure 5:**
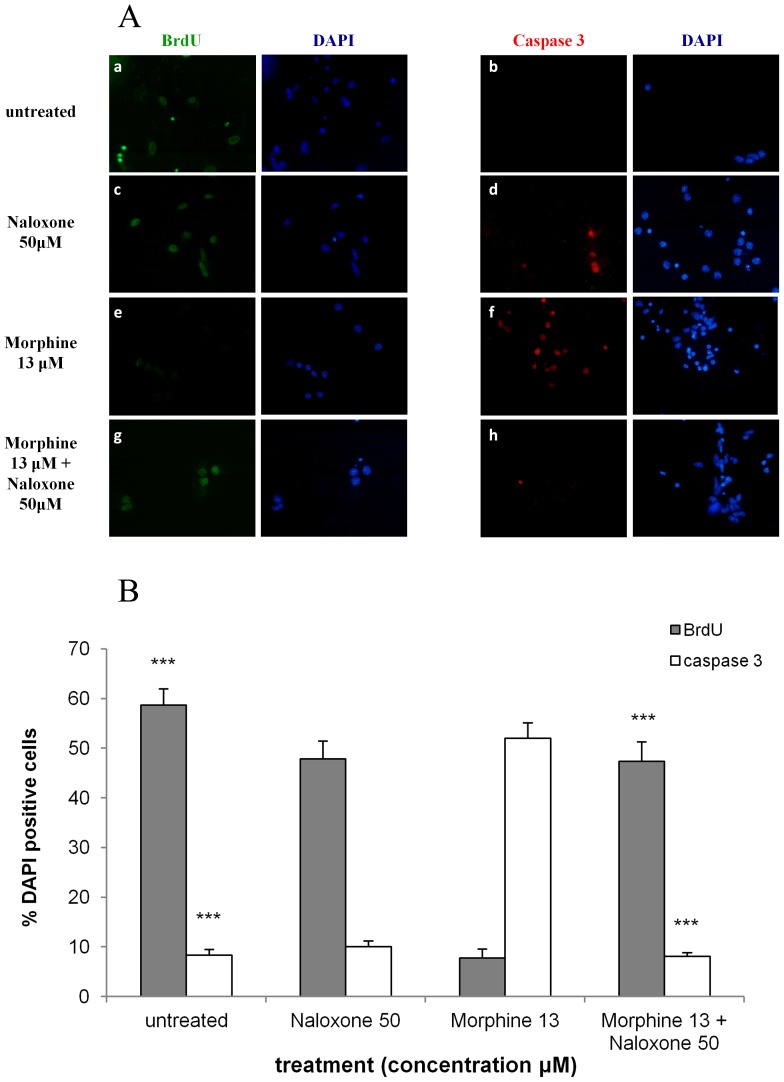
Naloxone reverses the anti-proliferative and pro-apoptotic effects of morphine on NPCs. Both the anti-proliferative and the pro-apoptotic effects of morphine were diminished by naloxone. The addition of naloxone to NPCs treated with morphine demonstrated a proliferative effect on NPCs and a reduced apoptotic effect compared with the morphine-treated groups (***p<0.001 vs. morphine 13 µM). Morphine 13 =  Morphine 13 µM; Naloxone 50 =  Naloxone 50 µM (**5A, 5B**).

To better understand the pharmacological pathway involved, we investigated whether the toxic effects of single dose morphine exposure was reversed by the opioid antagonist, naloxone. Untreated groups not exposed to morphine or naloxone had extensive BrdU incorporation (58.7±3.3%), while cells treated with 13 µM of morphine showed scant amounts of BrdU incorporation (7.8±1.8%). The addition of naloxone to morphine-treated cultures considerably attenuated (47.3±4.0%) but did not abolish completely the negative effect of morphine on cell proliferation (p<0.001) (Figure 5).

Moreover, naloxone also reversed the pro-apoptotic effects of morphine. As previously mentioned, the morphine treated cells (13 µM of morphine) demonstrated significant apoptosis (52.0±3.1%) compared with control values (8.3±1.2%). Here, the addition of naloxone to morphine-treated cultures entirely eliminated the pro-apototic effect of morphine (8.1±0.8%, p<0.001, compared with 13 µM morphine) with results similar to control values (Figure 5). This effect was dose dependent given that a smaller dose of naloxone also demonstrated a decrease in the number of apoptotic cells (data not shown). Naloxone, in itself, exhibited a minimal anti-proliferative/pro-apoptotic effect. BrdU incorporation and caspase-3 cell expression were near control values (47.8±3.7% BrdU- positive, 10.1±1.1% caspase-3- positive) in cultures exposed to naloxone alone.

### Naloxone reverses morphine's effect on neuronal differentiation and decreased nestin expression

**Figure 6:**
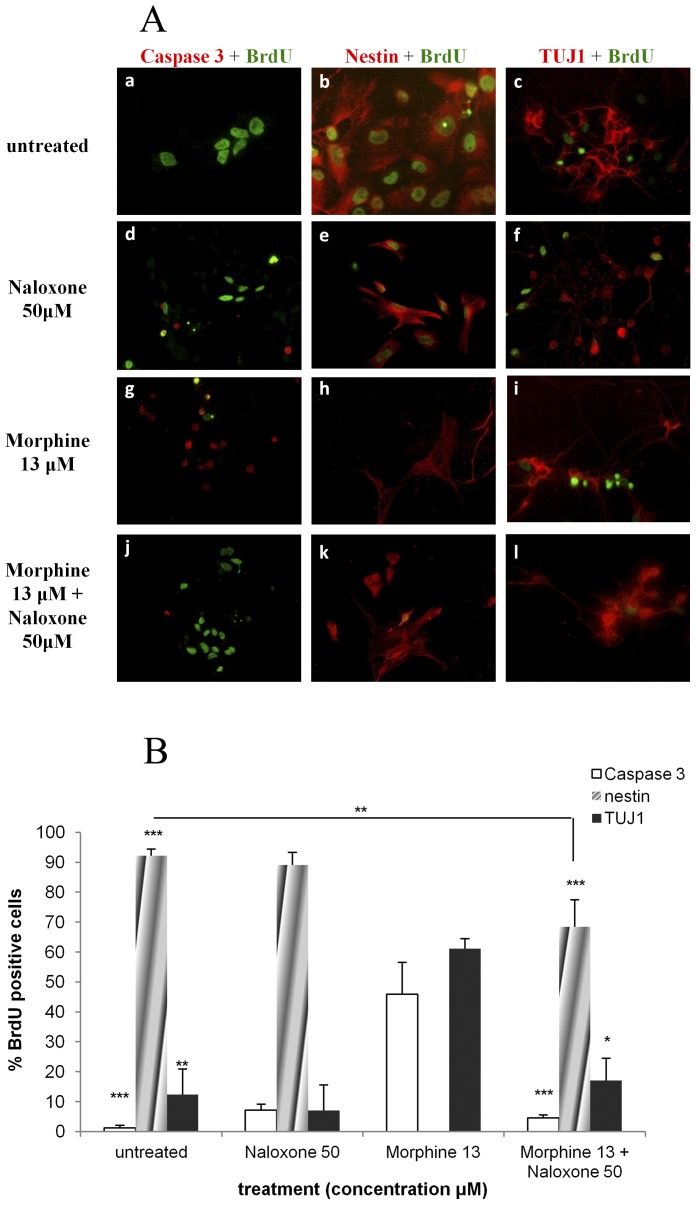
Naloxone reverses morphine increased-caspase-3 activity, neuronal differentiation and decreased NPC expression. Morphine results in increased apoptosis of BrdU- positive cells, decreases Nestin- positive- BrdU- positive cell expression and enhances neuronal differentiation as demonstrated in TUJ1- positive-BrdU- positive cell co-staining. Naloxone reverses these effects (*p<0.05, **p<0.01, ***p<0.001 vs. Morphine 13 µM). Morphine 13 =  Morphine 13 µM; Naloxone 50 =  Naloxone 50 µM (**6A, 6B**).

As mentioned previously, morphine reduced the number of proliferating cells expressing nestin. The percentage of NPCs incorporating BrdU and expressing nestin increased in the naloxone and morphine treated group (68.4±9.0%). These levels are lower than the levels of the untreated group (92.1±2.2%; p<0.01), but much higher than the zero values seen in the morphine-treated group. Naloxone also blocked the effects of morphine on NPC differentiation, causing a reduction in the number of TUJ1-expressing cells compared to those seen in controls (17.1±5.0% vs. 12.4±5.8%; Figure 6A, 6B).

### Naloxone decreases the number of apoptotic cells induced by morphine but does not affect their cell profile

**Figure 7:**
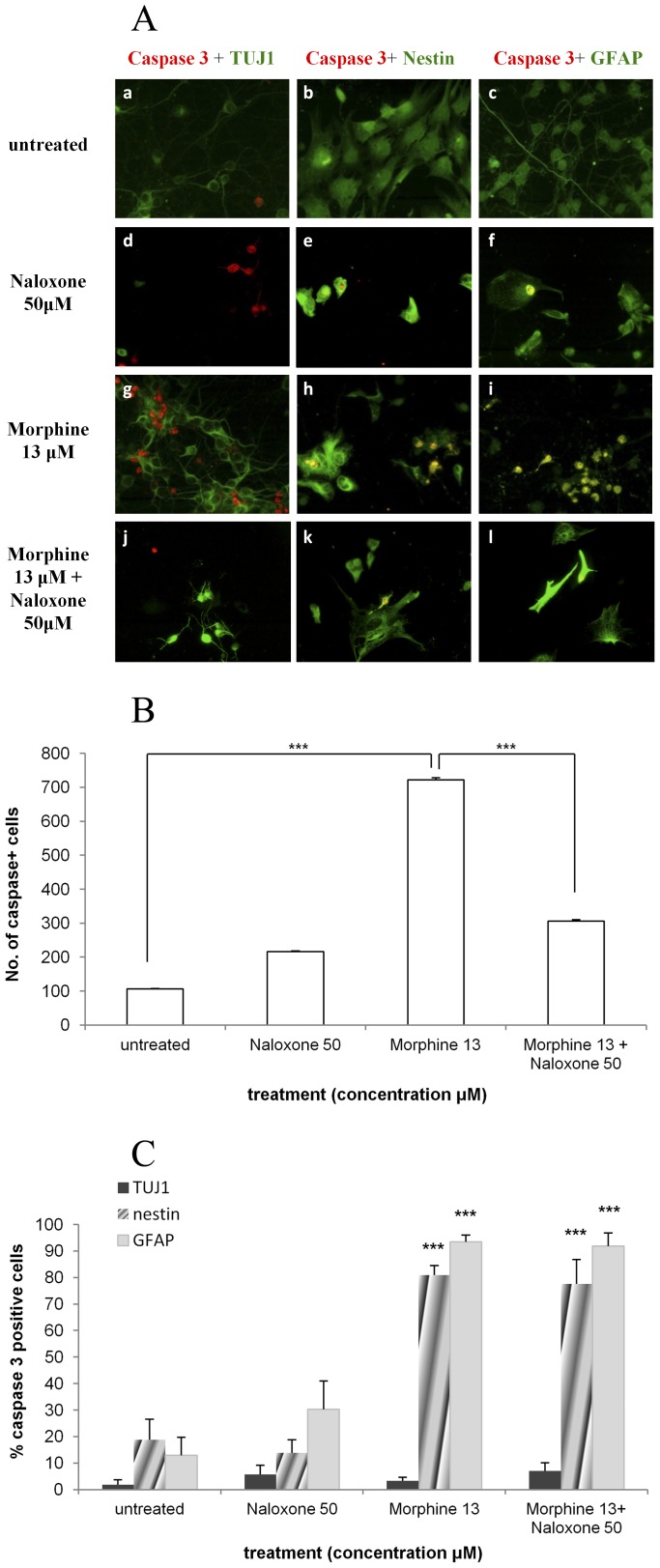
Naloxone decreases the number of apoptotic cells induced by morphine but does not affect their profile. Naloxone reduces the number of apoptotic NPCs induced by morphine (**7A**, **7B**) with no change in the profile of the dying cells (**7C**) (***p<0.001 vs. untreated). Morphine 13 =  Morphine 13 µM; Naloxone 50 =  Naloxone 50 µM.

The number of cells expressing active caspase-3 was 6.8 times greater in the 13 µM high-dose morphine treated group compared with the untreated group (722±6.1 cells vs. 106±1.4 cells; p<0.001). Addition of 50 µM naloxone to the morphine-treated cells reduced the number of apoptotic cells by half (306±3.8 cells; p<0.001 vs. morphine-treated cells; Figure 7A, 7B). The profile of the apoptotic cells remained unchanged by the addition of naloxone and the majority of caspase-3-expressing cells were nestin and GFAP but not TUJ1- positive (Figure 7A, 7C).

## Discussion

In this study we demonstrated the effect of a single, short term exposure of morphine on the fate of NPCs. Our findings show that the administration of a single dose of morphine *in vitro* induced inhibition of NPC proliferation and promoted apoptotic death in a dose dependent manner. A strong correlation was observed between these two opposing cell fate pathways of proliferation and death suggesting that proliferating cells are very susceptible to apoptosis. These effects were reversed with naloxone.

Though substantial research has investigated the impact of chronic opioid administration on neuronal development, very little focus has been placed in understanding whether a single, short term exposure to morphine has bearing on neuronal cell development. This is particularly crucial in the pregnant and pediatric population where opioids are regularly used for analgesia and very few equipotent drug substitutes exist. More importantly, even if opioids or some anesthetics have been reported to alter proliferation and differentiation of brain cells, when anesthesia and analgesia are considered necessary, it would be unethical to withhold these drugs. In their rapidly developing brain, the effect of any anesthetic or analgesic has the potential to have major negative implications. Chronic opioid administration has been shown to decrease neural stem cell (NSC) proliferation and suppresses glial cell growth, while inhibiting the generation of new neurons [9], [10], [13]. These observed effects appear to be primarily mediated via the opioid receptors [35], [36]. Chronic prenatal exposure to morphine has been shown to produce considerable somatosensory and cognitive deficits in mice [37]. Moreover, Hunt et al [38] demonstrated decreased cognitive dysfunction and social maturity in infants exposed to opioids in-utero. These results demonstrate the potential long-term cellular and behavioral changes caused by opioids on the developing brain.

On the other hand, very few studies exist with regard to the effects of a single dose morphine exposure *in vitro* or *in vivo* on neuronal development. Massa et al [39] demonstrated in an *in vivo* model that single bolus morphine did not induce apoptosis nor affect dendritic arbor differentiation or spinogenesis of pyramidal neurons during the peak synaptogenic period of the brain growth spurt in seven-to twenty- day old rat pups. Black et al [40] showed no change in active caspase-3 expression after a single exposure to morphine in 3 day old rat pups. Additionally, Emeterio et al [41] observed increased apoptotic cell death of neurons and glial cells of mature mice receiving chronic morphine treatment. However, caspase-3-positive cells were not detected following acute opioid administration. Spinal neural apoptosis was discerned in mature rats after chronic intrathecal morphine administration though none was observed after acute morphine administration. Furthermore, while certain evidence suggests that morphine has a destructive role in neuronal survival [42], [43] conflicting research support a possible neuroprotective role for morphine [44], [45]. Mouse primary cortical neurons cultured for 6 days with morphine demonstrated increased cell survival which was reversed by the addition of naloxone [32]. Additional evidence suggests that preconditioning with morphine induces neuroprotective effects in adult hippocampal brain slices [46]. Duhrsen et al [44] showed decreased neurological damage in postnatal rats pretreated with morphine in the presence of pain. On the other hand, in a study by McPherson et al [47], adult rat cognitive functioning was altered by repeated postnatal morphine treatment. Bajic et al [43] demonstrated distinct brain region sensitivity to the effects of morphine, with enhanced apoptosis in the cortex and amygdala, but no effect on the hippocampus, hypothalamus and periaquedactal gray area in neonatal rats exposed to repeated injections of morphine.

These results differ significantly from our findings. These differences can be explained by the fact that these studies were performed either during the early postnatal period or in mature rodents, an entirely different period of neuronal differentiation and development. Our study was performed during the embryonic period of brain development with neural progenitor cells clearly being more vulnerable to toxic insults and cell death. Our results are consistent with previous observations that show that opioid-induced signaling inhibits proliferation of neural progenitors *in vitro* [35]. Furthermore, Mei et al [48] demonstrated decreased spine density and morphology in pyramidal neurons of rats prenatally exposed to morphine. Clearly, the developmental stage of the neurons at the time of drug exposure, the duration of drug exposure and cell origin are a crucial determinant of the observed neurotoxicity. Further studies focused on the effects of morphine during the varied periods of pre- and post-natal brain development could help clarify these differing results.

Characterization of the apoptotic cells revealed contrasting results. While less than 20% of the apoptotic cells expressed nestin and a comparable percentage expressed GFAP in the untreated group, virtually a 100% of the apoptotic cells expressed both nestin and GFAP in the morphine treatment group (13 µM). This is most likely due to the expression of both markers by apoptotic cells in view of the fact that GFAP is an astrocytic marker, as well as neuroepithelial/radial glia marker [49], [50]. Interestingly, no significant differences were observed in the profile of the apoptotic neurons in the various treatment groups, implying considerable sensitivity of developing NPC cells to the neurotoxic effects of morphine, while at the same time relative tolerance of the immature neurons (TUJ1- positive cells) to a drug insult.

Dual expression of nestin and BrdU was shown to decrease in the morphine- treated groups, however, the levels of both active caspase-3 and TUJ1- positive cells from the proliferating (BrdU- positive) cells (50% and 60%, respectively) increased significantly in a dose dependent manner. These results imply that the fate of the majority of proliferating cells exposed to morphine is either to undergo apoptosis or forced early neuronal maturation. It can be assumed that after the death of the more sensitive nestin- positive cells exposed to morphine, the more “tolerant” immature neurons (TUJ1- positive cells) are also pre-destined to die. Alternatively, the stress of exposure to different concentrations of morphine may possibly trigger a cascade of cellular processes resulting eventually in enhanced differentiation [51], [52].

To better understand the mechanism by which morphine is anti-proliferative and pro-apoptotic, the opioid receptor antagonist, naloxone, was added to examine the hypothesis that morphine's effect is receptor mediated. Naloxone is a non-specific opioid receptor antagonist [53]. Opioid receptor expression has a distinct pattern of distribution in the nervous system of the prenatal rodent [54], [55]. The predominant receptor expressed during prenatal mouse brain development is the mu-opiod receptor. It is identified from embryonic day (E)12.5. Kappa-opioid receptors are first detected at E14.5 [56]. Delta-opioid receptors in the rodent brain are not expressed until postnatal day 10–14 [57]. Consequently, the primary action of the opioid system in the CNS in the early prenatal period and in our study is mediated through the mu-receptor. Moreover, while naloxone itself had minimal effect on proliferation or apoptosis of NPCs compared with the untreated group, the addition of naloxone to the morphine-treated culture did not result in a cumulative or synergistic effect but led to attenuation of both the anti-proliferative and the pro-apoptotic responses. These results imply a mu-receptor mediated mechanism for the effects of morphine on proliferation and apoptosis of NPCs.

Additionally, our work shows that morphine modified NPC differentiation towards a neuronal fate. Cellular proliferation in the murine cerebral cortex is known to occur during the late embryonic period (E12–E17) [58]. The combined potential modification in neuronal precursor cell fate may be immensely significant in the evolving mammalian brain. Taken together, these results may indicate a decrease in the self-renewal properties of the NPCs, in view of the fact that the proliferating (BrdU- positive) cells either undergo apoptosis or are forced to differentiate into young neurons thereby losing one of the unique traits that characterizes progenitor cells. This was attenuated, but not completely abolished, by the addition of naloxone. Opioids appear to modulate stem cell self-renewal and NPC differentiation by steering cells to a specific lineage [35], [36]. What this forced mass neuronal differentiation with consequential changes in the overall cell profile of the developing brain population implies is yet unclear and needs to be studied.

It is important to emphasize, that cells treated with naloxone alone, demonstrated findings similar to those of the untreated group suggesting that naloxone has minimal pro-apoptotic and anti-proliferative/differentiation properties in itself. The addition of naloxone to the morphine-treated cultures appears to “rescue” the neuroepithelial cells and impede the apoptotic process. The inhibition or stimulation of opioid receptors has been shown to affect the development and differentiation of neurons and glia [54]. The opioid receptor is clearly involved in the proliferation-differentiation balance of NPCs in the developing brain.

In conclusion, our findings indicate that even a single, short term exposure to morphine inhibits NPC proliferation, promotes apoptosis and alters NPC differentiation of isolated prenatal cerebral cortical cells *in vitro*. Though investigative evidence in animal models appears to be convincing, this certainly cannot be equated with humans. Clearly, the class of drug, dose, duration and timing of administration in relation to brain development appear to have a significant impact on the histological changes observed in these brains. More so, while *in vitro* studies are important, the impact of morphine on the developing brain *in vivo* and whether the changes observed *in vitro* correlate with long term cognitive and behavioral dysfunction must be further investigated and the subject of future studies. At the same time that researchers investigate the impact of opioids and their derivatives on human brain development, we must consider and explore alternative methods of providing pain relief to this population.
